# Physical activity and quality of life among breast cancer survivors: Pink SWAN

**DOI:** 10.1007/s00520-025-09156-8

**Published:** 2025-01-16

**Authors:** Brianna N. Leitzelar, Sybil L. Crawford, Beverly Levine, Kelly R. Ylitalo, Alicia B. Colvin, Kelley Pettee Gabriel, Gail A. Greendale, Nancy E. Avis

**Affiliations:** 1 Department of Social Sciences and Health Policy, Wake Forest University School of Medicine, Winston-Salem, NC USA; 2 Graduate School of Nursing, UMass Chan Medical School, Tan Chingfen Graduate School of Nursing, Worcester, MA USA; 3 Department of Public Health, Robbins College of Health and Human Sciences, Baylor University, Waco, TX USA; 4 Department of Epidemiology, School of Public Health, University of Pittsburgh, Pittsburgh, PA USA; 5 Department of Epidemiology, School of Public Health, The University of Alabama at Birmingham, Birmingham, AL USA; 6 Department of Medicine, Division of Geriatrics, University of California Los Angeles, Los Angeles, CA USA

**Keywords:** Breast neoplasms, Cancer, Quality of life, Physical activity, Survivorship

## Abstract

**Purpose:**

To describe physical activity (PA) trajectories across 10 years post-breast cancer diagnosis and examine their association with quality of life (QoL).

**Methods:**

Participants from the longitudinal Study of Women’s Health Across the Nation who developed incident breast cancer completed the Quality of Life in Adult Cancer Survivors scale (QLACS) which has 12 domains. Breast cancer survivors (BCS) with at least one post-diagnosis measure of the Kaiser Physical Activity Survey (PA) were included (*n* = 96). We estimated metabolic equivalents of task minutes per week (MET-min/week) for the two most frequent sport/exercise activities. Group-based trajectory modeling determined PA trajectories over 10 years post-diagnosis. Analysis of covariance assessed associations between PA trajectory group and the three QLACS domains with the worst scores (fatigue, pain, and recurrence-related distress), adjusted for PA and other relevant covariates.

**Results:**

There were four post-diagnosis PA trajectories: consistently very low/no PA (“inactive,” 11.5%); consistently some, but below aerobic PA guideline (“below guideline,” 48.9%); generally met aerobic PA guideline with a slight decline (“met guideline,” 22.2%); and exceeded aerobic PA guideline (“exceeded guideline,” 18.8%). In adjusted models, the below guideline group reported more fatigue than the met or exceeded groups and more pain than the met guideline group, but there were no group differences in recurrence-related distress.

**Conclusion:**

The majority of BCS did not meet the aerobic PA guideline over 10 years post diagnosis. BCS who met the aerobic PA guideline reported less fatigue and pain compared to those who did not meet the guideline in adjusted analyses, suggesting a negative association between PA and QoL.

**Supplementary Information:**

The online version contains supplementary material available at 10.1007/s00520-025-09156-8.

## Introduction

Breast cancer is the leading cancer in terms of incidence among females in the United States [1]. Given excellent survival rates (5-year: 91%; 10-year: 85%), there are more than 3.8 million breast cancer survivors (BCS) in the United States [1]. Long-term and late effects of breast cancer and its treatment include cardiotoxicities, lasting symptoms (e.g., fatigue and pain), and psychosocial concerns (e.g., recurrence-related distress and financial concerns) [2]. In addition, many BCS report decreased quality of life (QoL) following diagnosis [3–5], with a subset experiencing impaired QoL long-term (i.e., up to 10 years post-diagnosis) [4, 5]. In light of the large number of BCS and lasting negative effects, supportive care efforts to mitigate the persisting effects of cancer and its treatment are important.

The American Cancer Society and American College of Sports Medicine previously published evidence-based PA guidelines for cancer survivors [6, 7]. Identical to those developed for the general public [8], guidelines include 150–300 min of moderate- or vigorous- aerobic PA and at least 2 days of muscle strengthening activity per week, while also minimizing daily sitting time. Cross-sectional [9–11] and longitudinal research [12–19], focused largely on aerobic PA, has previously demonstrated a positive association between PA and QoL among BCS. These studies, however, lack diverse samples [12–15], include mainly short-term BCS (< 5 years of diagnosis) [9–11] or have relatively short follow-up periods (6 months to 3 years) [15–19].

The Study of Women’s Health Across the Nation (SWAN) provided the opportunity to address these gaps in a longitudinal cohort of BCS. The objectives of the present analyses were to (1) describe PA trajectories among BCS up to 10 years post-diagnosis and (2) examine the associations between post-diagnosis PA trajectories and QoL, adjusted for relevant covariates.

## Methods

### Participants and procedures

SWAN is a multi-racial/ethnic cohort study characterizing biological and psychosocial changes occurring during the menopause transition [20]. From 1995 to 1997, seven clinical sites recruited participants. All sites recruited non-Hispanic White women for approximately half of their sample. In addition, each site recruited women from one of four underrepresented racial/ethnic groups within the geographical area of the recruiting site (Black, Chinese, Hispanic, or Japanese women). All sites used a common protocol that was approved by each site’s Institutional Review Board. All participants provided written informed consent.

Eligibility criteria for SWAN included the following: age 42–52 years at enrollment; an intact uterus and at least one ovary; not pregnant, lactating, using oral contraceptives or hormone therapy; and a menstrual cycle in the 3 months before screening. In-person assessments were conducted at baseline and approximately annually through follow-up visit 15, which occurred in 2015–2016. Measures included medical, reproductive, and menstrual history; lifestyle and psychosocial characteristics; physical and psychological symptoms; and anthropometrics. Participants chose to complete instruments in Cantonese, English, Japanese, or Spanish.

Pink SWAN *BCS* were 151 women who developed *incident breast cancer* following SWAN enrollment and had no other prior cancer. To verify self-reported diagnosis and treatment, a medical oncologist adjudicated medical records from women reporting incident breast cancer. Medical records were available for 73% of the BCS, and agreement between medical records and self-reported diagnosis was high (94.5% agreement). As we have previously described [4], a questionnaire assessing treatment-related factors and QoL was mailed to BCS who were active in SWAN at visit 15 (*N* = 130); 109 responded (83.8%). The present analyses included these 109 BCS.

### Measures

#### Primary outcome

The primary outcome for these analyses was the Quality of Life in Adult Cancer Survivors (QLACS) Scale, which was administered to BCS at SWAN visit 15 as part of the one-time Pink SWAN questionnaire. The QLACS is a 47-item QoL questionnaire designed for adult cancer survivors [21]. The measure contains seven general QoL domains that do not refer to cancer that assess cognitive problems, fatigue, negative feelings, physical pain, positive feelings, sexual problems, and social avoidance and five cancer-specific domains that include appearance concerns, cancer-related benefits, family-related distress, financial problems, and recurrence-related distress. Items are rated on a 7-point Likert scale ranging from 1 “Never” to 7 “Always,” and responses are summed to generate 12 domain-specific scores. Higher scores represent more of the domain studied (e.g., more fatigue and more pain) and hence poorer QoL, with the exception of positive feelings and cancer-related benefits where higher scores represent better QoL. The QLACS had good internal consistency and construct validity among short- and long-term cancer survivors in previous studies [21, 22].

#### Physical activity

PA was assessed at SWAN baseline and visits 3, 5, 6, 9, 12, 13, and 15 using the valid and reliable Kaiser Physical Activity Survey (KPAS; [23]). The KPAS assesses self-reported PA in multiple domains: household and caregiving (e.g., chores and meal preparation), active living (i.e., biking/walking for transportation, watching TV), and sports/exercise. We quantified PA using the sports/exercise domain, given its alignment with the aerobic PA guideline for cancer survivors. For the sports/exercise domain, BCS reported up to two most frequent sport or exercise activities on a typical week over the past year, the frequency, and the duration for each reported sport or exercise type. Open-ended responses to the sports/exercise activities were categorized into one of 65 coded physical activities and assigned an absolute metabolic equivalent of task (MET) value based on the 2011 Compendium of Physical Activities [24], as previously described [25]. One MET is equivalent to 3.5 mL of oxygen per kilogram per minute, which is equivalent to the energy expended during quiet sitting. We calculated MET-mins/week using metabolic equivalents multiplied by the reported duration of the activity [25] and used a threshold of 450 MET-min/week (3.0 MET × 150 min/week) to categorize the sample as achieving the aerobic PA guideline or not [8, 25].

#### Covariates

Potential covariates considered for our ANCOVA models included factors hypothesized or demonstrated to be significantly related to QoL in previous literature [3–5]. Covariates included variables that were measured at multiple SWAN visits (time-variant) and those measured at only one visit (time-invariant). To account for the potential impact of either the most proximal value or an aggregate measure of time-varying covariates, we considered these covariates in two ways: (1) the value of the covariate at visit 15 and (2) a post-diagnosis summary measure of the variable.

The following describes the time-varying covariates. Body mass index (kg/m^2^), perceived stress [26], and self-reported physical function (Medical Outcomes Short Form Physical Functioning Scale [27]) were included as continuous variables. Categorical variables were alcohol consumption (< 1 drink per month vs. 1–8 drinks/month, > 8 drinks/month), anxiety symptoms (low vs. high anxiety based on a cutoff score of 4) [28], any systemtic hormone use (yes vs. no), financial hardship (very/somewhat hard vs. not at all hard to pay for basics), menopausal status (pre- or perimenopausal vs. post-menopausal due to surgery, post-menopausal due to natural reasons, unknown due to hormone use, missing), menopausal hormone therapy (yes vs. no to any non-breast cancer-related serotonin-estrogen reuptake inhibitors), partner status (married or living as married vs. not married), number of comorbidities (0 vs. 1, > 2), smoking status (never vs. past/current), sleep problems (difficulty falling asleep, staying asleep, and/or early morning awakening ≥ 3 times/week in the past 2 weeks, yes vs. no) [29], and self-assessed health (very good/excellent vs. good, fair/poor).

Post-diagnosis summary measures of time-varying variables were created as follows (all summary measures also include visit 15 data). For continuous measures (BMI, perceived stress, and self-reported physical function), the summary measure was an average across post-diagnosis visits. For categorical variables, we used the proportion of post-diagnosis vists meeting variable-specific criteria. These included high alcohol consumption (consuming > 8 drinks per month); experiencing high anxiety; ever using systemic hormones; experiencing any financial hardship; being pre- or peri-menopausal; reporting menopausal hormone therapy; reporting being married; reporting > 2 comorbidities; not currently smoking; experiencing any sleep problems; and reporting poor self-assessed health.

Continuous time invariant measures included age at visit 15 and age at breast cancer diagnosis. Categorical time invariant measures included educational attainment (< college vs. > college degree), self-identified race/ethnicity (Black, Chinese, Hispanic, Japanese, and non-Hispanic White), SWAN site, cancer stage at diagnosis (0, I, II/III, unknown or missing), ever chemotherapy (yes/no), ever endocrine therapy (yes/no based on receipt of any of the following drugs: Nolvadex/tamoxifen, Arimidex/anastrozole, Femara/letrozole, Aromasin/exemestane, Evista/raloxifene, Faslodex/fuvestrant, Fareston/toremifen, other), ever radiation therapy (yes/no), and surgery (any/none).

### Statistical analyses

We characterized the study sample using descriptive statistics (i.e., means, variance, and frequencies). Missing data in models were handled differently according to the specific procedure, as noted below. To describe 10-year post-diagnosis PA trajectories (objective 1), we conducted group-based trajectory modeling using SAS PROC TRAJ (SAS version 9.4) to identify subgroups of BCS who followed similar PA trajectories over time. PROC TRAJ assumes missing data are missing completely at random (MCAR) [30]. Analyses modeled PA as a function of time since diagnosis from 0 to 10 years post-diagnosis. We evaluated models with 2–5 trajectory groups and included linear and quadratic terms. Objective criteria for model selection included improvement in the Bayesian information criteria [31] and group size of at least 10% of the sample assigned, as well as subjective judgement regarding meaningful differences in trajectories. Posterior probability (i.e., highest estimated probability of group membership for the individual) determined trajectory group assignment for each BCS. Our main trajectory analysis was based on all participants with at least one post-diagnosis measure of PA. We also conducted a sensitivity trajectory analysis that included only those participants with at least 2 post-diagnosis measures of PA.

To examine the associations between PA trajectory group and QLACS domains (objective 2), we first tested bivariate associations between PA trajectory group and each QLACS domain using separate Kruskal–Wallis’ analysis of variance (ANOVA) due to non-normal outcome variables (QLACS domain scores) (R version 4.2.3). We then determined bivariate associations between QLACS domain scores and covariates (time-invariant variables, visit 15 value of time-variant variables, and post-diagnosis summary of time-variant variables) using Pearson correlations (continuous variables) or ANOVAs (categorical variables). Covariate criteria for inclusion in ANCOVA models included a significant association with the QLACS domain studied (*p* < 0.10). If both versions of time-varying covariates met criteria, we selected the version associated with the QLACS outcome with the lowest *p*-value to avoid multicollinearity of predictors.

ANCOVA models done using R (version 4.2.3) first included only the closest pre-diagnosis measure of PA without other covariates to determine if pre-diagnosis PA affected the observed bivariate associations between PA trajectory group and QLACS domains scores (“Adjusted Model 1”). We next added SWAN site and other relevant covariates to a backward selection model (“Adjusted Model 2”) using the R function “step.” “Step” sequentially removes variables from the full model and selects the best model using the Akaike Information Criterion. Sensitivity analysis for covariate selection using lasso regression [32] were consistent (data not shown). Final models included only covariates that met the *p* < 0.10 criteria. We checked model assumptions and used heteroscedasticity-consistent estimators to account for non-constant error variance when present in the models. Sensitivity analysis included the removal of influential outliers using Belsley et al.’s method [33]. These analyses all used a complete-case approach to handle missing data.

## Results

### Sample characteristics

Of the 109 BCS who returned the questionnaire at SWAN follow-up visit 15, 96 had at least one measure of post-diagnosis PA and thus constituted our analytic sample. BCS included in the present analyses were diagnosed at earlier stages than BCS who were excluded (*X*^2^ = 43.6, *p* < 0.001); there were no other significant differences. Among the 96 BCS in our analyses, *n* = 24 (25%) had only one measure of post-diagnosis PA included in the trajectory modeling, due to being diagnosed closer in time to visit 15 when the QLACS was administered. The median number of post-diagnosis PA data points among BCS in our sample was between two and three (modal value three), with 20% of the sample having five or more PA data points (data not shown.)

BCS were predominantly non-Hispanic White (53%) or Black (24%), completed college or higher (54%), and had early-stage (63% stage 0/I) breast cancer at diagnosis. Their mean age at diagnosis was 56.7 (sd = 6.1) years and their mean age at Pink SWAN survey completion was 66.5 (sd = 3.0) years (Table 1). Walking was the most frequently reported sports/exercise activity (*n* = 53), followed by muscle strengthening activities (e.g., Pilates, weight bearing exercise and leg exercise) (*n* = 8), bicycling/spinning (*n* = 7), and yoga or stretching (*n* = 7).

**Table 1:** Sample characteristics

	*n*	Mean (sd) or frequency
Age at Pink SWAN survey (years)	96	66.5 (3.0)
Age at breast cancer diagnosis (years)	96	56.7 (6.1)
Time between diagnosis and QLACS completion (years)	96	9.3 (5.5)
Pre-diagnosis physical activity^a^ (MET-mins/week)	95	466.9 (529.7)
Body mass index (V15) (kg/m^2^)	80	28.5 (6.7)
SF-36 Physical Function (V15)	88	77.9 (24.2)
Perceived stress (V15)	91	6.4 (2.6)
Race/ethnicity		
Black	23	24.0%
Chinese	8	8.3%
Hispanic	2	2.1%
Japanese	12	12.5%
Non-Hispanic White	51	53.1%
Education		
College degree or higher	52	54.7%
Less than college	43	45.3%
Missing	1	
Partner status (V15)		
Married or living as married	62	65.3%
Not married or living as married	33	34.7%
Missing	1	
Financial strain (V15)		
No	66	69.5%
Yes	29	30.5%
Missing	1	
Alcohol consumption (V15)		
< 1 drink per month	43	48.9%
1–8 drinks per month	25	28.4%
> 8 drinks per month	20	22.7%
Missing	8	
Smoking status (V15)		
Current or past smoker	34	39.1%
Never smoker	53	60.9%
Missing	9	
Sleep problems (V15)		
No	50	54.9%
Yes	41	45.1%
Missing	5	
Anxiety symptoms (V15)		
Low	81	93.1%
High	6	6.9%
Missing	9	
Self-assessed health		
Excellent or very good	39	42.9%
Good	38	41.8%
Fair or Poor	14	15.4%
Missing	5	
Number of comorbidities (V15)		
0	8	8.8%
1	19	20.9%
> 2	64	70.3%
Missing	5	
Hormone category (V15)		
None	59	64.8%
Non-systemic hormones	4	4.4%
Non-breast cancer related selective estrogen receptor modulators	2	2.2%
Breast cancer-related selective estrogen receptor modulators	6	6.6%
Antineoplastic hormones	20	22.0%
Missing	5	
Menopause status (V15)		
Natural post-menopause	86	94.5%
Surgical menopause	5	5.5%
Missing	5	
Cancer stage at diagnosis		
0	24	25.0%
I	46	47.9%
II/III	17	17.7%
Unknown or missing	9	9.4%
Cancer treatment-surgery (*n*, % yes)	95	99.0%
Cancer-treatment-chemo (*n*, % yes)	34	35.4%
Cancer treatment-radiation (*n*, % yes)	70	72.9%
Ever Endocrine therapy^b,c^ (*n*, %yes)	61	63.5%

Percentages do not include missing values in the denominator

*SF-36* medical outcomes short form, *V15* value of variable at SWAN visit 15

^a^Closest pre-diagnosis measure of physical activity measured using the Kaiser Physical Activity Survey

^b^Yes/no based on receipt of any of the following drugs: Nolvadex/tamoxifen, Arimidex/anastrozole, Femara/letrozole, Aromasin/exemestane, Evista/raloxifene, Faslodex/fuvestrant, Fareston/toremifen, and other

^c^*n* = 12 missing

### Physical activity trajectories

**Fig. 1:**
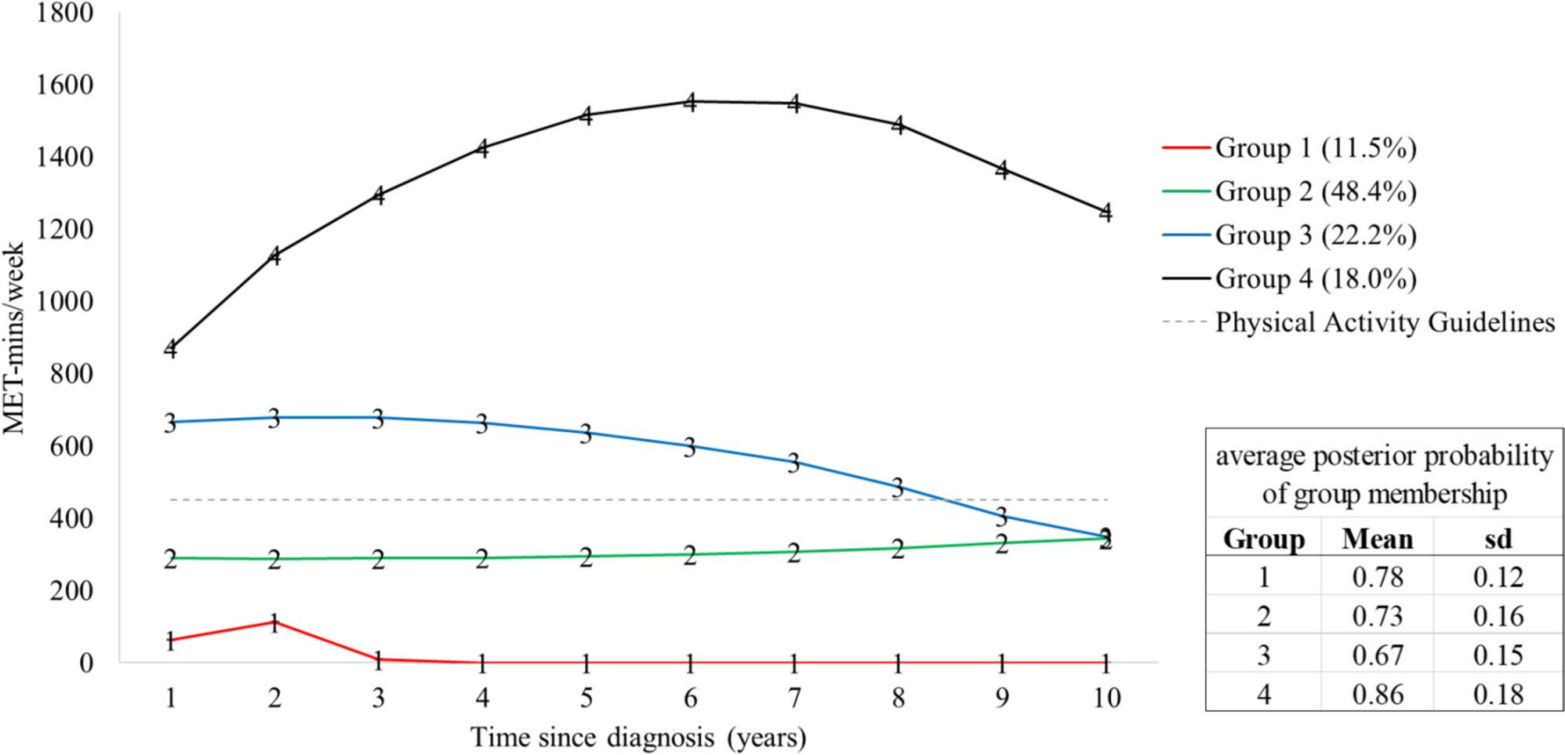
Predicted mean values of physical activity by trajectory group over 1 to 10 years post-diagnosis among BCS. MET-mins/week = metabolic equivalent of task in minutes per week

We identified four post-diagnosis PA trajectories and assigned women to the group for which they had the maximum posterior probability (Fig. 1). Across all of the years since diagnosis time points on the *X*-axis in the figure below, there were at least 10 data points; for years 1, 2, 3, and 4 following diagnosis, there were more than 25 data points.

The following descriptions include predicted values from the group-based trajectory analysis. Group 1 (11.5%, *n* = 9) was characterized by being “inactive,” with consistently very low/no PA across all 10 years post-diagnosis. This group engaged in an estimated average of only 63 MET-mins per week (95% CI: 0–356) at 1-year post-diagnosis and decreased to 0 MET-mins/week (95% CI: 0–352) by year 3. Group 2 (48.4%, *n* = 55), the largest group, consistently reported some PA, but their mean amount never reached the level of the aerobic PA guideline (“low active”). Their average PA was 305 MET-min/week (95% CI: 0–843) across the 10-year period. Group 3 (22.2%, *n* = 14) met the aerobic PA guideline and had an average of 667 MET-mins/week (95% CI: 0–2349) at 1-year post-diagnosis (“met guideline”). They consistently met the aerobic PA guideline until about year 9 when they appeared to dip below the guideline. Group 4 (18.0%, *n* = 18.8) reported high PA, with an average of 870 MET-min/week (95% CI: 418–1322) at 1-year post-diagnosis, with an evident increase until year 6 (“exceeded guideline”). Although their PA appeared to decrease after year 6, this group, on average, still exceeded the aerobic PA guideline at year 10 (mean PA: 1248 MET-mins/week; 95% CI: 837–1659).

Due to the timing of their cancer diagnosis relative to the administration of the QLACS survey, 24 women (25%) contributed only a single post-diagnosis physical activity (PA) data point to the trajectory group analysis. We conducted sensitivity analyses excluding these 24 women, and found that the optimal number of trajectory groups, as well as the shape and location of the trajectories, remained consistent with the results from the full sample of 96 women (data not shown). Therefore, we retained the trajectory analysis for the full sample.

### Associations between PA trajectories and QoL domains

Fatigue (mean score = 12.18), sexual problems (mean score = 10.8), physical pain (mean score = 10.1), recurrence-related distress (mean score = 9.55), and cognitive problems (mean score = 9.33) were the most problematic QLACS domains (Table 2). Fatigue, pain, and recurrence-related distress were also associated with PA trajectory group in bivariate analyses (*p* < 0.05) (Table 2). Although the average domain scores for sexual and cognitive problems were also high (i.e., problematic), these domains were not significantly associated with PA trajectory group. Multivariable modeling was therefore limited to fatigue, pain, and recurrence-related distress.

**Table 2:** Means and standard deviations of QLACS domains in the total sample and by post-diagnosis aerobic physical activity trajectory group

QLACS domain	Total Sample		Group 1		Group 2		Group 3		Group 4		*p*^a^
			“Inactive”		“Low active”		“Met guideline”		“Exceeded guideline”		
	*n* = 96		*n* = 9		*n* = 55		*n* = 14		*n* = 18		
	Mean	sd	Mean	sd	Mean	sd	Mean	sd	Mean	sd	
General domains											
Negative feelings	9.24	3.65	8.44	2.74	9.82	3.70	9.50	4.77	7.67	2.45	0.173
Positive feelings	21.79	4.84	21.89	4.34	20.76	5.36	23.07	4.21	23.89	2.76	0.138
Cognitive problems	9.33	4.41	6.81	1.55	10.00	5.14	9.21	3.93	8.61	2.52	0.184
Sexual problems	10.81	5.46	9.78	3.23	10.67	6.23	10.43	5.69	12.06	3.35	0.294
Physical pain	10.10	5.55	8.67	4.66	11.60	6.08	8.00	4.96	7.83	2.87	**0.030**
Fatigue	12.18	5.67	10.22	3.67	13.70	5.89	11.07	6.63	9.39	3.24	**0.019**
Social avoidance	7.69	4.86	5.70	2.39	8.56	5.21	6.79	5.09	6.72	4.14	0.158
Cancer-specific domains											
Appearance concerns	6.89	4.73	5.67	2.29	7.80	5.13	5.86	4.74	5.54	3.92	**0.030**
Financial problems	5.49	3.47	4.33	0.71	6.00	3.81	5.36	4.52	4.59	1.50	0.422
Recurrence-related distress	9.55	4.90	7.44	3.54	11.00	5.29	7.57	2.98	7.59	3.99	**0.007**
Family-related distress	7.00	4.57	6.33	5.48	8.15	5.08	5.43	2.24	5.06	2.44	**0.048**
Benefits	15.07	7.51	13.44	6.77	14.91	7.42	15.00	7.52	16.44	8.46	0.869

QLACS domain scores range from 4 to 28, except for family-related distress, which ranges from 3 to 21. Higher scores indicate more of the domain studied. Bold text indicates significant bivariate association between PA trajectory group and QLACS domain score (*p* < 0.05)

^a^Result from Kruskal–Wallis analysis of variance testing the bivariate association between PA trajectory group membership and QLACS domain

We were interested in comparing groups with low PA to groups with high PA. Although Group 1 (the inactive group) had low PA, we did not include this group in further analyses due to the small sample size (*n* = 9) and concern that statistical power would not be sufficient to detect differences. We also considered combining groups; however, this option would lose much of the information gained by the group-based trajectory analysis. Combining only Group 1 with Group 2 was also considered, but these two groups, however, engage in very different behaviors (none versus low levels of PA). Further, each PA trajectory group had distinctly different QLACS domain scores across several domains and combining groups would dilute any potential group differences. Given these considerations, we ultimately decided to exclude Group 1 (the inactive group) from further analyses. Group 2, the “low active” group served as the reference group, because it provided the opportunity to compare BCS with low PA to BCS with higher PA levels.

#### Fatigue

In both the unadjusted model and Model 1 adjusting for pre-diagnosis PA, the low active group reported significantly more fatigue compared to BCS who exceeded the aerobic PA guideline (*p* < 0.01 for both models; Table 3). After adjusting for relevant covariates (Adjusted Model 2), low active BCS reported significantly more fatigue than BCS who met or exceeded the guideline by 4.1 and 3.8 points, respectively (*p* < 0.05). Results were consistent in sensitivity analyses removing two influential outliers (Table S1, Online Resource 1).

**Table 3:** Linear regression models of QLACS fatigue as a function of post-diagnosis physical activity trajectory group

	*Unadjusted* (*n* = 87)		*Adjusted Model 1*^*a*^ (*n* = 86)		*Adjusted Model 2*^*b*^ (*n* = 78)	
	Beta estimate	*p*	Beta estimate	*p*	Beta estimate	*p*
Met guideline vs. low active	− 2.62	0.121	− 2.85	0.104	** − 4.11**	**0.018**
Exceeded guideline vs. low active	** − 4.30**	**0.006**	** − 5.12**	**0.009**	** − 3.74**	**0.039**
Pre-diagnosis MET-mins/week			0.00	0.554	0.00	0.429
Perceived stress (V15)					0.68	0.004
SF-36 Physical Function (V15)					− 0.07	0.010
Chemotherapy (yes vs. no)					3.76	0.003

Bold text indicates significant PA trajectory group difference in QLACS fatigue (*p* < 0.05). Higher QLACS scores indicate more of the domain studied. Except for pre-diagnosis physical activity, only covariates which reached significance at the *p* < 0.05 level are listed in the table, and covariates significant to the *p* < 0.01 level are described in their associated notes

^a^Adjusted Model 1 includes pre-diagnosis physical activity (closest pre-diagnosis measure)

^b^Adjusted Model 2 also includes SWAN site

#### Pain

BCS who engaged in low levels of PA reported significantly more pain compared to those who met or exceeded the guideline for aerobic PA in the unadjusted model and Model 1 adjusting for pre-diagnosis PA (*p* < 0.05 for both models; Table 4). After adjusting for relevant covariates, BCS in the low active group had significantly more pain, by an estimated 4.1 points, than those who met the guideline (*p* = 0.003) (Adjusted Model 2). Sensitivity analyses removing three outliers did not substantially alter the results (Table S2, Online resource 1).

**Table 4:** QLACS physical pain as a function of post-diagnosis physical activity trajectory group

	*Unadjusted* (*n* = 87)		*Adjusted Model 1*^*a*^ (*n* = 86)		*Adjusted Model 2*^*b*^ (*n* = 78)	
	Beta estimate^**c**^	*p*^**c**^	Beta estimate^**c**^	*p*^**c**^	Beta estimate^**c**^	*p*^**c**^
Met guideline vs. low active	− 3.62	**0.027**	− 3.66	**0.040**	− 4.11	**0.003**
Exceeded guideline vs. low active	− 3.78	**0.001**	− 3.91	**0.031**	− 1.84	0.307
Pre-diagnosis MET-mins/week			0.00	0.925	0.00	0.289
SF-36 Physical Function (V15)					− 0.13	< 0.001

Bold text indicates significant PA trajectory group difference in QLACS pain (*p* < 0.05). Higher QLACS scores indicate more of the domain studied. Except for pre-diagnosis physical activity, only covariates which reached significance at the *p* < 0.05 level are listed in the table, and covariates significant to the *p* < 0.01 level are described in their associated notes

^a^Adjusted Model 1 includes pre-diagnosis physical activity (closest pre-diagnosis measure)

^b^Adjusted Model 2 also includes ever chemotherapy, average post-diagnosis perceived stress, and SWAN site

^**c**^Model results corrected to account for non-constant error variance using heteroscedasticity-consistent estimators

#### Recurrence-related distress

Low active BCS had significantly more recurrence-related distress compared to those who met or exceeded the guideline in the unadjusted model and Model 1, adjusting for pre-diagnosis PA (*p* < 0.05 for both models; Table 5). This relationship was not significant in the model adjusting for all relevant covariates. As there were no influential outliers in this model, we did not conduct sensitivity analyses.

**Table 5:** QLACS recurrence-related distress as a function of post-diagnosis physical activity trajectory group

	*Unadjusted* (*n* = 86)		*Adjusted Model 1*^*a*^ (*n* = 85)		*Adjusted Model 2*^*b*^ (*n* = 80)	
	Beta estimate^**c**^	*p*^**c**^	Beta estimate^**c**^	*p*^**c**^	Beta estimate^**c**^	*p*^**c**^
Met guideline vs. low active	− 3.43	**0.002**	− 3.63	**0.004**	− 1.55	0.282
Exceeded guideline vs. low active	− 3.41	**0.007**	− 3.81	**0.044**	− 2.79	0.131
Pre-diagnosis MET-mins/week			0.00	0.600	0.00	0.162
Cancer stage (I vs. 0)					2.18	0.069
Cancer stage (II/III vs. 0)					5.28	0.017
Cancer stage (unknown/missing vs. 0)					0.07	0.960
Self-assessed health (fair/poor vs. very good/excellent) (V15)					3.43	0.115
Self-assessed health (good vs. very good/excellent) (V15)					2.47	0.068

Bold text indicates significant PA trajectory group difference in QLACS recurrence-related distress (*p* < 0.05). Higher QLACS scores indicate more of the domain studied. Except for pre-diagnosis physical activity, only covariates which reached significance at the *p* < 0.05 level are listed in the table, and covariates significant to the *p* < 0.01 level are described in their associated notes

^a^Adjusted Model 1 includes pre-diagnosis physical activity (closest pre-diagnosis measure)

^b^Adjusted Model 2 also includes SWAN site

^c^Model corrected to account for non-constant error variance using heteroscedasticity-consistent estimators

## Discussion

The present study examined long-term post-diagnosis PA trajectories and their association with post-diagnosis QoL among BCS. We found four distinct PA trajectories among BCS over 10 years post diagnosis with the majority of BCS reporting some PA, but not enough PA to meet the established guideline for aerobic PA. BCS engaging in low amounts of PA reported more fatigue and pain than those who met the aerobic PA guideline. Low active BCS also reported more recurrence-related distress than those who met or exceeded the guideline in unadjusted analyses, but not when adjusted for cancer stage and self-reported health.

Previous investigations have also reported distinct post-diagnosis PA trajectories among BCS [34–37]. For example, Lucas et al. [34] found 3 PA trajectories among BCS; 42% were consistently below PA guidelines, 46% consistently met PA guidelines, and 12% consistently exceeded PA guidelines across 10 months post-diagnosis. While the majority of BCS in our sample also did not change, we did find a small group (the exceeded group), who started with very high PA and increased over the first 5 years post-diagnosis. We also found that the exceeded and met guideline groups began to decline at about 5 years post-diagnosis. Three other studies also identified groups of BCS whose PA changed over the first 1–2 years post-diagnosis (either increased or decreased) [35–37], though there is variation in the number and patterns of PA trajectories. Variations are likely due to differences in sample sizes, sample characteristics (e.g., age and time since diagnosis), and/or time course of measurement (1–2 years vs. 10 years), but overall, studies found that approximately 40–60% of BCS did not meet PA guidelines over the measurement period [34–37]. In the present sample, 60% of participants were consistently below the aerobic PA guideline, suggesting inactive BCS may be likely to remain inactive for up to 10 years post-diagnosis.

Our finding that lower PA was associated with greater fatigue is largely consistent with previous research. Findings from previous cross-sectional studies have also demonstrated a significant association between higher levels of PA and less fatigue among BCS on average 7–10 years post-diagnosis [38, 39]. In addition, low post-diagnosis PA predicted fatigue 6 months to 5 years later [12, 18, 40] in prospective studies. The present study, however, is the first to our knowledge to examine PA patterns across 10 years to predict subsequent fatigue while accounting for potential confounding factors (chemotherapy, perceived stress, and physical function). However, neither the present study, nor previously mentioned studies, can determine causality in the associations between fatigue and PA. Recent meta-analyses have concluded that exercise interventions are effective for reducing cancer-related fatigue [41, 42], suggesting that PA engagement leads to lower fatigue levels among BCS. Proposed mechanisms for the effect of exercise on cancer-related fatigue span biological (e.g., reduced inflammation), behavioral (e.g., improved sleep), and psychological (e.g., reduced anxiety and/or depression) domains [43]. However, since our analyses were cross-sectional, we cannot rule out the possibility that greater fatigue may have contributed to lower PA. BCS often report fatigue, characterized by low energy and persistent tiredness, as a significant barrier to engaging in PA [44]. Overcoming these feelings of ongoing exhaustion can be particularly challenging and prevent BCS from engaging in day-to-day activities, including PA.

We found that BCS who met the aerobic PA guideline reported less pain at 10 years post-diagnosis than BCS who engaged in low levels of PA. This relationship held, even when accounting for pre-diagnosis PA and other relevant factors (chemotherapy, perceived stress, and self-reported physical function). These results are consistent with another longitudinal study that found that BCS who met PA guidelines at 5 and 10 years post diagnosis reported less pain at 10 years post diagnosis than BCS who were inactive [45]. One prospective study did not find an association between PA and pain across approximately 3 years [12], but their pain measure assessed arm and breast pain/sensations, which may not be affected by aerobic PA. Although our results suggest that BCS who are physically active across 10 years post-diagnosis report less pain, causality cannot be determined due to the cross-sectional nature of our analyses. Meta-analytic evidence suggests that exercise training may have a small-to-moderate beneficial effect on pain among people living with cancer [46]. However, this evidence is limited by significant reporting bias, heterogeneity in the measures used, and the inclusion of various pain etiologies (e.g., musculoskeletal and neuropathic). PA is thought to influence pain through several mechanisms, including enhanced self-efficacy, improved sleep, better mood, and potential biological changes to neuroendocrine systems and inflammatory processes [47–49]. Nevertheless, more specific research on cancer-related pain mechanisms is needed [46, 47].

The present study is among the few to examine PA in relation to recurrence-related distress [50–53]. We found that low active BCS reported more recurrence-related distress than BCS who met or exceeded the aerobic PA guideline, but this difference was not significant in analyses adjusting for other relevant covariates (cancer stage, self-assessed health). This suggests that PA may be related to recurrence-related distress, but later stage at diagnosis and lower self-assessed health among BCS not meeting the aerobic PA guideline may account for their higher recurrence-related distress. Findings from previous studies are equivocal. Among studies of mixed or other cancer types, two studies found more PA was associated with less recurrence-related worry/fear [50, 51], and two others found no significant association [52, 53]. We propose that the association between PA and recurrence-related distress is bidirectional. On one hand, engaging in PA may reduce risk of cancer recurrence [54], which can give BCS a sense of control over future cancer risk and subsequently reduce distress about potential recurrence. On the other hand, the experience of recurrence-related might influence engagement in health promoting behaviors, including PA. BCS may be motivated to adopt health-promoting behaviors as a way to cope with their distress; conversely, those who are overwhelmed by distress might avoid engaging in such behaviors.

It is relevant to note that we did not include the least active BCS (Group 1) in the multivariable models of PA trajectory group and fatigue, pain, and recurrence-related distress due to limited sample size concerns with statistical power. Interestingly, BCS accumulating little to no PA over 10 years post-diagnosis was interesting because they reported QLACS scores similar to BCS meeting or exceeding the aerobic PA guideline. One hypothesis is that these BCS were accumulating PA in other domains, such as household or active living activities, outside of the sports/exercise domain that we used for the present analysis. To better understand these women’s PA behavior, we conducted exploratory analyses on all PA domains measured by the KPAS (i.e., household and caregiving and active living) as well as on pre-diagnosis PA levels. We found that all four groups engaged in similar levels of household and caregiving and active living tasks and that pre-diagnosis PA followed the same pattern as post-diagnosis PA (Table S3, Online Resource 1). Thus, it seems unlikely that the PA domains outside of the sports/exercise domain contribute to the QLACS scores of Group 1. Investigating the differences between BCS reporting little to no sports/exercise-related PA and low-active BCS and BCS meeting or exceeding the aerobic PA guideline may provide valuable insights into the diverse PA behaviors of BCS and their implications for long-term QoL.

Some limitations of these analyses are noted. The average posterior probability of group membership for Group 3 (“met guideline”) of 0.67 was just below the suggested cut off for reliability (0.70) and we can only be moderately confident that BCS were accurately classified into Group 3. Further, 25% of our sample contributed only a single post-diagnosis PA data point to the trajectory analysis; however, our sensitivity analysis excluding these participants did not alter the shape nor location of the trajectories. We also noted that PROC TRAJ assumes missing data are MCAR, which is the simplest assumption to make and often fails to hold. However, in this situation, missing PA data likely is not correlated either physical activity or with QoL, but rather with the timing of onset of breast cancer relative to when the QLACS was administered. Therefore, the MCAR assumption may be reasonable here. Further, the small number of BCS in Group 1 resulted in excluding this group from the multivariable analyses. Due to the cross-sectional design and observational nature of the data, we were not able to determine directionality in our study. Cancer-related symptoms and concerns are frequently reported barriers to PA [44], and it is likely that high levels of fatigue, pain, and/or recurrence-related distress contribute to low levels of PA. Future studies should focus on investigating the mechanisms underlying the associations between PA and QoL (fatigue, pain, and recurrence-related distress) to understand their complex nature.

The present analysis has a number of strengths. The sample was followed longitudinally up to 10 years post-diagnosis providing the opportunity to characterize longer-term PA trajectories than other studies. In addition, the use of a validated measure of self-reported PA and standardized method to calculate MET-mins/week provides a more nuanced model of PA trajectories. Finally, a comprehensive list of potential covariates allowed us to investigate the association between PA trajectory group and QoL accounting for potential explanatory factors.

### Clinical implications

Our findings show that the majority of BCS in this study exhibited consistently low levels of PA over 10 years post-diagnosis. This suggests the need for regular PA assessment and discussions concerning PA recommendations during patient-provider visits. The association between meeting the established guideline for aerobic PA reported and better QoL (i.e., less fatigue, pain, and recurrence-related distress) suggests a link between PA and QoL, but we are not able to inform the direction of the association. However, existing meta-analyses of exercise interventions show that structured exercise programs can reduce fatigue and pain, albeit with small-to-moderate effects [41, 42, 46]. Conversely, symptoms such as fatigue and pain often act as barriers to PA engagement [44], indicating a bidirectional association. Interventions to reduce fatigue and pain may help BCS to become more physically active. Providers should, therefore, consider patients’ individual experiences with QoL and symptoms, as well as their current PA levels to provide personalized guidance. We suggest that guidance which simultaneously manages symptoms and encourages PA promotion will be the most beneficial for the overall well-being of BCS.

## Supplementary Information

Below is the link to the electronic supplementary material.

Supplementary file1 (PDF 153 KB)

## Data Availability

SWAN provides access to public use datasets that include data from SWAN screening, the baseline visit and follow-up visits (https://agingresearchbiobank.nia.nih.gov/). To preserve participant confidentiality, some, but not all, of the data used for this manuscript are contained in the public use datasets. A link to the public use datasets is also located on the SWAN web site: http://www.swanstudy.org/swan-research/data-access/. Investigators who require assistance accessing the public use dataset may contact the SWAN Coordinating Center at the following email address: swanaccess@edc.pitt.edu.
