# Supplementary material for: Physical activity and quality of life among breast cancer survivors: Pink SWAN
Source: Support Care Cancer. 2025 Jan 16;33(2):101. doi: 10.1007/s00520-025-09156-8 (PMC11735529; doi:10.1007/s00520-025-09156-8)
Supplement: Supplementary file 1 — Supplementary file1 (PDF 153 KB) [file 520_2025_9156_MOESM1_ESM.pdf]

Article Title: Physical Activity and Quality of Life Among Breast Cancer Survivors: Pink SWAN

Journal Name: Supportive Care in Cancer

Author Names: Brianna N. Leitzelar, Sybil Crawford, Beverly Levine, Kelly R. Ylitalo, Alicia B. Colvin, Kelley Pettee Gabriel, Gail A. Greendale, Nancy E. Avis

Corresponding Author: Brianna N. Leitzelar
email: leitz025@umn.edu

Online Resource 1

Table S1. Sensitivity analysis for the ANCOVA model of QLACS fatigue as a function of post-diagnosis physical activity trajectory group

	Beta estimate	*p*
Met guidelines vs. Below guidelines	**-5.12**	**0.007**
Exceeded guidelines vs. Below guidelines	**-3.75**	**0.033**
Pre-diagnosis MET-mins/week	0.00	0.419
Perceived Stress (V15)	0.75	0.002
SF-36 Physical Function (V15)	-0.08	0.005
Chemotherapy (yes vs. no)	3.28	0.007

Note. Model results are based on sensitivity analyses removing two influential outliers from the dataset; total sample size = 76. Bold text indicates significant PA trajectory group difference in QLACS fatigue ($p<0.05$). Higher QLACS scores indicate more of the domain studied. Except for pre-diagnosis physical activity, only covariates which reached significance at the $p<0.05$ level are listed in the table.

Table S2. Sensitivity analysis for the ANCOVA model of QLACS physical pain as a function of post-diagnosis physical activity trajectory group

	Beta estimate[a]	*p*[a]
Met guidelines vs. Below guidelines	-3.36	**0.006**
Exceeded guidelines vs. Below guidelines	-1.12	0.503
Pre-diagnosis MET-mins/week	0.00	0.498
SF-36 Physical Function (V15)	-0.13	<0.001

Note. Model results are based on sensitivity analyses removing three influential outliers from the dataset; total sample size = 75. Bold text indicates significant PA trajectory group difference in QLACS pain ($p<0.05$). Higher QLACS scores indicate more of the domain studied. Except for pre-diagnosis physical activity, only covariates which reached significance at the $p<0.05$ level are listed in the table.

[a]Model results corrected to account for non-constant error variance using heteroscedasticity-consistent estimators

Table S3. Means and standard deviations of physical activity behavior at visit 15 and pre-diagnosis physical activity by post-diagnosis physical activity trajectory group

	Group 1 **"Inactive"**			Group 2 **"Low active"**			Group 3 **"Met Guideline"**			Group 4 **"Exceeded guideline"**		
	n	mean	sd	n	mean	sd	n	mean	sd	n	mean	sd
Active living	9	1.6	0.4	50	2.0	0.8	9	2.6	0.8	18	2.5	0.7
Household and caregiving	9	2.2	0.8	50	2.4	0.8	11	2.4	0.9	18	2.3	0.8
Sport/Exercise	8	1.8	0.7	50	2.5	0.9	11	3.4	0.7	18	4.1	0.4
Total physical activity score	8	5.8	1.3	50	6.9	1.5	9	8.3	1.1	18	9.0	1.0
Pre-diagnosis MET-mins/week	9	48.8	65.4	55	307.7	416.4	14	586.7	419.8	17	1104.5	536.4
Post-diagnosis MET-mins/week	8	102.7	290.4	50	283.2	350.2	11	640.9	339.1	18	1331.3	493.0

Note. Active living, Household and caregiving, sport/exercise, and total physical activity scores were obtained from the Kaiser Physical Activity Survey (KPAS). Pre- and post-diagnosis MET-mins/week are derived from the sports/exercise data on the KPAS using procedures outlined in the methods section of our manuscript as well as in Ylitalo et al (2022).
